# Network-Assisted Systems Biology Analysis of the Mitochondrial Proteome in a Pre-Clinical Model of Ischemia, Revascularization and Post-Conditioning

**DOI:** 10.3390/ijms23042087

**Published:** 2022-02-14

**Authors:** Alex Gallinat, Gemma Vilahur, Teresa Padró, Lina Badimon

**Affiliations:** 1 Cardiovascular Program-ICCC, IR-Hospital Santa Creui Sant Pau, IIB-Sant Pau, 08041 Barcelona, Spain; agallinat@santpau.cat (A.G.); gvilahur@santpau.cat (G.V.); Tpadro@santpau.cat (T.P.); 2 CIBERCV-Instituto de Salud Carlos III, 28029 Madrid, Spain; 3 Cardiovascular Research Chair, UAB, 08193 Barcelona, Spain

**Keywords:** post-conditioning, ischemia, cardioprotection, mitochondria, proteomics, network biology

## Abstract

Infarct size is the major risk predictor for developing heart failure after an acute myocardial infarction (AMI). The discovery of the conditioning phenomena (i.e., repetitive brief cycles of ischemia applied either before or after a prolonged ischemic insult) has highlighted the existence of endogenous protective mechanisms of the heart potentially limiting infarct size after revascularization. However, most cardioprotective strategies, aiming at infarct size reduction, have failed in clinical studies. Thus, cardioprotection is an unmet clinical need. In the present study, we took a network-assisted systems biology approach to explore the mitochondrial proteomic signature of the myocardium after ischemia, ischemia with direct revascularization, and ischemia with re-establishment of blood flow by post-conditioning in a swine model of AMI. Furthermore, network extension with the ENCODE project human regulatory data allowed the prediction of potential transcription factors at play in the response to post-conditioning of the myocardium. Collectively, our results identify cardiac metabolism as a driver of cardioprotection, highlighting a dual role for post-conditioning promoting metabolic reprogramming of the myocardium, and a protective response mediated by VDAC2 and DJ-1 in the mitochondria.

## 1. Introduction

Despite a drop in mortality of acute myocardial infarction (AMI) in the past decades due to the application of primary percutaneous coronary intervention (PPCI) [1,2], AMI survivors are at high risk of developing heart failure (HF) [3,4], which is a cause of a high morbidity and mortality worldwide [5] and of huge global burden on healthcare and economic resources [6]. AMI is an ischemic event caused by the sudden interruption of coronary blood supply to the myocardium. From the onset of AMI, a wave-front of necrosis grows within the at-risk myocardium [7]. Due to the minimal regenerative capacity of the adult human heart, there is no recovery of the lost functional tissue after an AMI [8] and thus, infarct size is the major predictor of AMI clinical outcomes [9,10]. The early and successful restoration of coronary blood flow by PPCI is the most effective strategy to limit infarct size. However, this process that occurs within the very first minutes of reflow paradoxically has the potential to exacerbate damage and accounts for a significant part of the final infarct size [11,12]. A vast number of cardioprotective therapies aimed at reducing infarct size have failed to demonstrate clinical benefits [13,14]. Thus, cardioprotection is currently an unmet clinical need. A better understanding of the mechanisms at play in the ischemic myocardium is needed in order to both explore novel cardioprotective strategies and to improve translation.

In 1986, Murray et al. [15] first described how the application of repetitive brief cycles of ischemia and revascularization prior to a prolonged ischemic insult greatly limits infarct size and improves post-AMI heart function. This practice was called ischemic pre-conditioning and has demonstrated the existence of endogenous cardioprotective mechanisms that are worth exploring. However, the clinical application of pre-conditioning is limited because AMI is a sudden event and cannot be accurately predicted. Later on, Zhao et al. [16] reported that ischemic conditioning also results in significant protection when applied at the time of the re-establishment of coronary blood flow after a prolonged ischemic insult. This practice was called post-conditioning, and markedly reduced infarct size, endothelial dysfunction, and post-ischemic blood flow defects [16,17,18]. Despite broadly proven benefits of ischemic conditioning in animal models, it turned controversial in clinical trials [19,20,21], possibly due to differences in the conditioning protocols, patient medications, and comorbidities [22,23,24]. Thus, a better understanding of the mechanisms at play is needed to improve translation. Many studies have focused on the identification of single molecules and apoptosis-related pathways at play [25], but a global characterization of the coordinated changes in the myocardium following post-conditioning is still lacking.

Network medicine is a rapidly growing field combining systems biology with network science to understand the relationship between molecular signatures and disease pathogenesis [26,27]. Based on the assumption that biological processes are not primarily constrained by single proteins or discrete pathways, but rather a network of complex molecular interactions, network medicine provides powerful tools to understand the driving mechanisms of disease [28].

Mitochondria play a central role in the generation and maintenance of cellular ATP pools. They also provide a hub for cell metabolism, programmed cell death, and have multiple roles in signaling. These features make them central organelles for cellular adaptation to ischemia and revascularization, and for cardioprotection [29,30,31]. Nearly all identified signaling pathways in the cardioprotective response triggered by the conditioning phenomena converge in the mitochondria; thus, mitochondria are believed to be the effector organelles for cardioprotection [32].

The present work is the continuation of a previous study in which we took a proteomic approach to identify regulators of post-conditioning and their signaling pathways [33]. In the first part of the study, a beneficial effect of post-conditioning over the global heart function, infarct size, and cell survival was found (Supplemental Figure S1), and an effect on the canonical aryl-hydrocarbon receptor pathway was shown [33]. Now, we have investigated whether a network-assisted systems biology approach focusing on the mitochondrial proteome evolution all along ischemia, revascularization, and post-conditioning, would shed additional light on the understanding of the molecular basis of cardioprotection beyond signaling, and how post-conditioning modifies the response of the myocardium to the re-establishment of coronary blood flow.

## 2. Results

### 2.1. Ischemia, Revascularization, and Post-Conditioning Impacts on the Swine Mitochondrial Proteome

**Figure 1:**
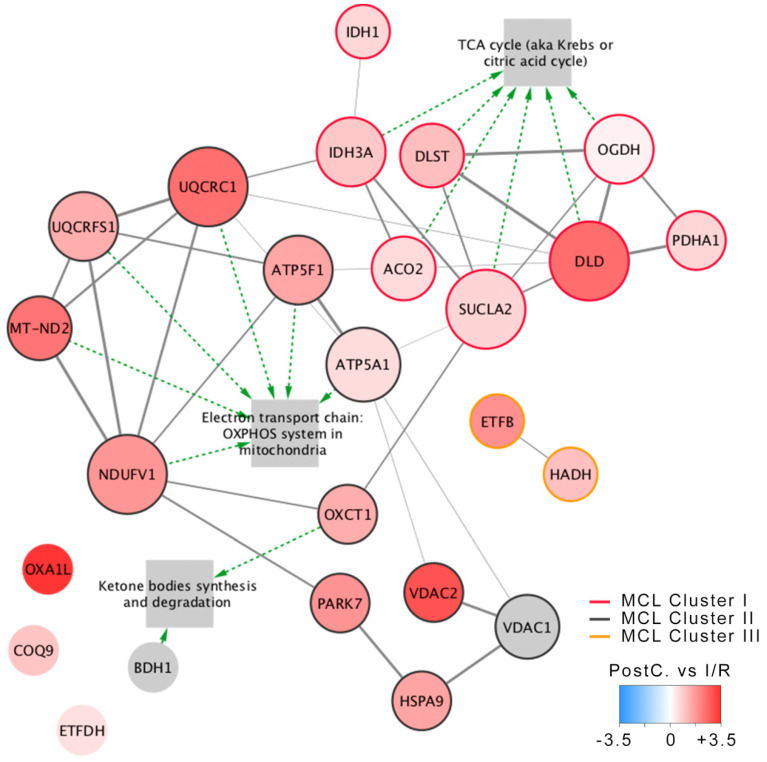
Mitochondrial physical PPI network analysis. All proteins detected to be differentially expressed (absolute logFC > 0.5) in at least one condition were included. PPIs were retrieved from the STRING database, and the Markov clustering (MCL) strategy with an inflation parameter of 1.4 was applied to identify protein-protein interaction (PPI) clusters. The PPI network of differentially regulated proteins, including the most relevant enriched pathways, was detected in the analysis. Green arrows connect pathways with their matching proteins in the network. Node color represents the logFC between post-conditioning and direct revascularization, size represents node degree within the network, and edge thickness the confidence of interaction between nodes. Abbreviations—I/R: Ischemia/revascularization group; PostC: Post-conditioning group; ACO2: Heart aconitase; ATP5A1: ATP synthase subunit alpha; ATP5F1: ATP synthase beta subunit; BDH1: D-beta-hydroxybutyrate dehydrogenase; COQ9: Ubiquinone biosynthesis protein COQ9; DLD: Dihydrolepolyl dehydrogenase; DLST: Dihydrolipollysin-residue succinyltransferase component of 2-oxoglutarate dehydrogenase complex; ETFB: Electron transfer flavoprotein subunit beta; ETFDH: Electron transfer flavoprotein-ubiquinone oxidoreductase; HADH: Hydroxyacyl-coenzyme A dehydrogenase; HSPA9: Stress-70 protein, mitochondrial; IDH2: Isocitrate dehydrogenase [NADPH]; IDH3A: Isocitrate dehydrogenase [NAD] subunit alpha; MT-ND2: NADH-ubiquinone oxidoreductase; NDUFV1: NADH dehydrogenase [ubiquinone] flavoprotein 1; OGDH: 2 oxoglutarate dehydrogenase; OXA1L: Mitochondrial inner membrane protein; OXCT1: Succinyl-CoA:3-ketoacid-coenzyme; PARK7: Protein DJ-1; PDHA1: Pyruvate dehydrogenase; SUCLA2: Succinyl-CoA ligase [ADP-forming] subunit beta; UQCRC1: Cytochrome b-c1 complex subunit 1; UQCRFS1: Cytochrome b-c1 complex subunit Rieske; VDAC1: Voltage-dependent anion-selective channel protein 1; VDAC2: Voltage-dependent anion-selective channel protein 2.

The proteomic characterization of the myocardium at risk revealed at least 26 mitochondrial proteins to be differentially regulated as a result of ischemia, revascularization, and post-conditioning in the swine heart (Supplemental Table S1). In order to depict an overall functional characterization, we built a physical protein–protein interaction (PPI) network according to the STRING [34] database with 26 differentially regulated proteins detected across all conditions (Figure 1). The resulting network revealed a significant PPI enrichment, indicating that the differentially expressed proteins are biologically connected as a group [26]. Then, we applied the Markov clustering (MCL) [35,36] strategy and ran a functional overrepresentation analysis upon Gene Ontology biological process terms and pathways from the Wikipathways [37] database. The MCL algorithm identified three PPI-enriched clusters within the network, tightly correlating with the significantly overrepresented pathways. More specifically, the tricarboxylic acid (TCA) cycle (WP78; FDR ≈ 0) and the oxidative phosphorylation (OXPHOS) system (WP111; FDR ≈ 0) (Figure 1—see Supplemental Table S2 for a complete list of the overrepresented pathways). Accordingly, strongly significant enrichments were found for Gene Ontology biological process terms related to the energy metabolism and cellular respiration (Table 1), collectively highlighting the pivotal role of the energy metabolism in organ resilience during ischemia and revascularization.

### 2.2. Tricarboxylic Acid Cycle

**Figure 2:**
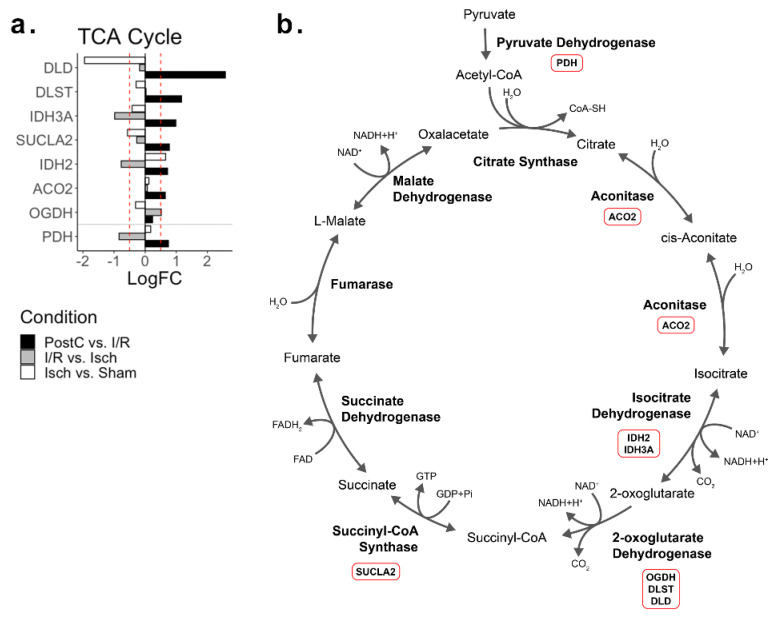
Tricarboxylic acid (TCA) cycle related differentially regulated proteins. (**a**) LogFC plot of TCA cycle related proteins across ischemia, reperfusion, and post-conditioning. The red dashed line indicates the threshold at absolute logFC > 0.5. (**b**) TCA cycle pathway scheme with the differentially regulated proteins detected highlighted within red boxes. Abbreviations—Isch: Ischemia group; I/R: Ischemia/revascularization group; PostC: Post-conditioning group; ACO2: Heart aconitase; DLD: Dihydrolepolyl dehydrogenase; DLST: Dihydrolipollysin-residue succinyltransferase component of 2-oxoglutarate dehydrogenase complex; IDH2: Isocitrate dehydrogenase [NADPH]; IDH3A: Isocitrate dehydrogenase [NAD] subunit alpha; OGDH: 2 oxoglutarate dehydrogenase; PDHA1: Pyruvate dehydrogenase; SUCLA2: Succinyl-CoA ligase [ADP-forming] subunit beta.

A total of 8 proteins involved in the TCA cycle were detected to be differentially regulated across all conditions (Figure 2). In ischemic hearts, both the succinyl-CoA ligase (SUCLA2) and the dihydrolepolyl dehydrogenase (DLD), a component of the oxoglutarate dehydrogenase complex, were found to be down-regulated. After the re-establishment of coronary blood flow, there was no recovery of SUCLA2 or DLD, but instead a further down-regulation of the TCA cycle member isocitrate dehydrogenase subunit alpha (IDH3A) and the isocitrate dehydrogenase 2 (IDH2) was detected. The oxoglutarate dehydrogenase complex component OGDH was up-regulated after revascularization. When post-conditioning was performed, a wide up-regulation of enzymes involved in the progression of the TCA cycle was detected, with the recovery of IDH3A and all the oxoglutarate dehydrogenase complex members. Heart aconitase (ACO2), the enzyme responsible for the first two reactions of the TCA cycle, was up-regulated following post-conditioning.

### 2.3. Oxidative Phosphorylation

**Figure 3:**
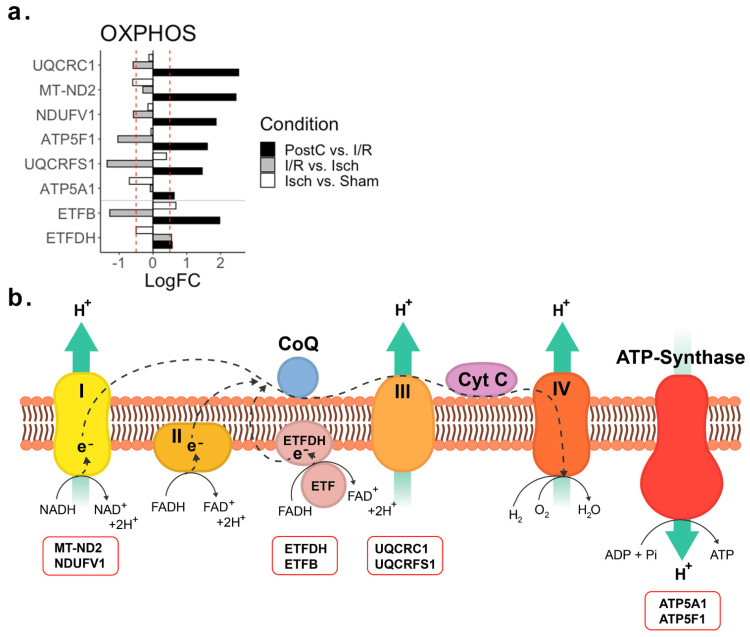
Oxidative phosphorylation (OXPHOS)-related differentially regulated proteins. (**a**) LogFC plot of the OXPHOS related proteins across ischemia, revascularization, and post-conditioning. The red dashed line indicates the threshold at absolute logFC > 0.5. (**b**) OXPHOS scheme with the differentially regulated proteins detected highlighted within red boxes. Abbreviations—Isch: Ischemia group; I/R: Ischemia/ revascularization group; PostC: Post-conditioning group; ATP5A1: ATP synthase subunit alpha; ATP5F1: ATP synthase beta subunit; ETFB: Electron transfer flavoprotein subunit beta; ETFDH: Electron transfer flavoprotein-ubiquinone oxidoreductase; MT-ND2: NADH-ubiquinone oxidoreductase; NDUFV1: NADH dehydrogenase [ubiquinone] flavoprotein 1; UQCRC1: Cytochrome b-c1 complex subunit 1; UQCRFS1: Cytochrome b-c1 complex subunit Rieske.

A total of 6 proteins belonging to the electron transport chain complexes I, III, and V were affected by ischemia, direct revascularization, and post-conditioning (Figure 3). Ischemic hearts exhibited a down-regulation of proteins belonging to both complexes I and V. After the re-establishment of coronary blood flow, further down-regulations of complex I and V were seen, together with a strong down-regulation of the complex III core catalytic subunit (cytochrome b-c1 oxidoreductase complex subunit Rieske; UQCRFS1). Conversely, post-conditioned hearts exhibited a strong up-regulation of the electron transport chain complexes I, III, and V. Additionally, the electron-transfer flavoprotein oxidoreductase (ETFDH) and the electron-transfer flavoprotein subunit beta (ETFB), which are not part of the electron transport chain complexes themselves but a crucial link between the fatty acids beta-oxidation and the oxidative phosphorylation, were also up-regulated in the post-conditioned hearts.

### 2.4. Non Metabolic Proteins

**Figure 4:**
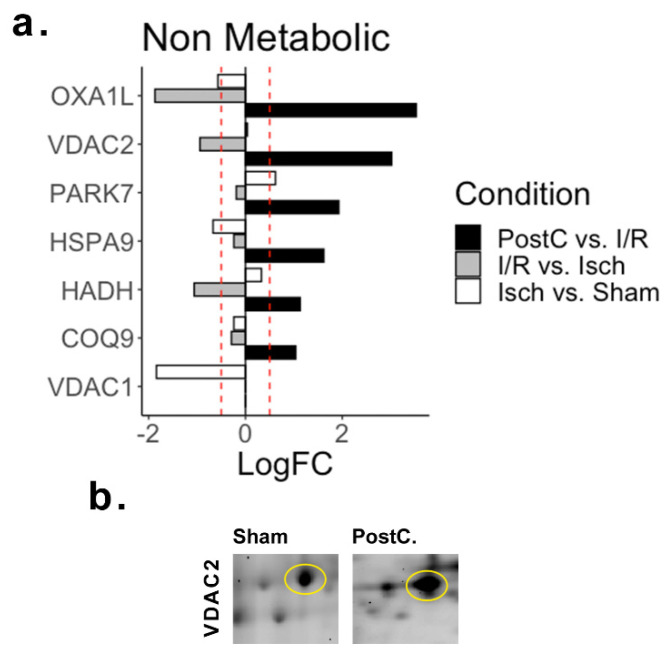
Non-metabolic differentially regulated proteins. (**a**) LogFC plot of the non-metabolic proteins detected across ischemia, reperfusion, and post-conditioning. The red dashed line indicates the threshold at absolute logFC > 0.5. (**b**) Detail of VDAC2 spot across conditions. Abbreviations—Isch: Ischemia group; I/R: Ischemia/revascularization group; PostC: Post-conditioning group; COQ9: Ubiquinone biosynthesis protein COQ9; HADH: Hydroxyacyl-coenzyme A dehydrogenase; HSPA9: Stress-70 protein, mitochondrial; OXA1L: Mitochondrial inner membrane protein; PARK7: Protein DJ-1; VDAC1: Voltage-dependent anion-selective channel protein 1; VDAC2: Voltage-dependent anion-selective channel protein 2.

Between the differentially regulated proteins detected across all conditions, seven proteins with non-metabolic functions were detected to be strongly up-regulated following post-conditioning (Figure 4). Amongst them, we found that the mitochondrial inner membrane protein (OXA1L), the voltage-dependent anion-selective channel 2 (VDAC2), and protein DJ-1 (PARK7) were greatly up-regulated. Also, the mitochondrial stress-70 protein (HSPA9, also known as mortalin), which is functionally related to PARK7, was found to be up-regulated in the post-conditioned hearts.

### 2.5. Prediction of Ischemic Post-Conditioning Mitochondrial Regulatory Network

**Figure 5:**
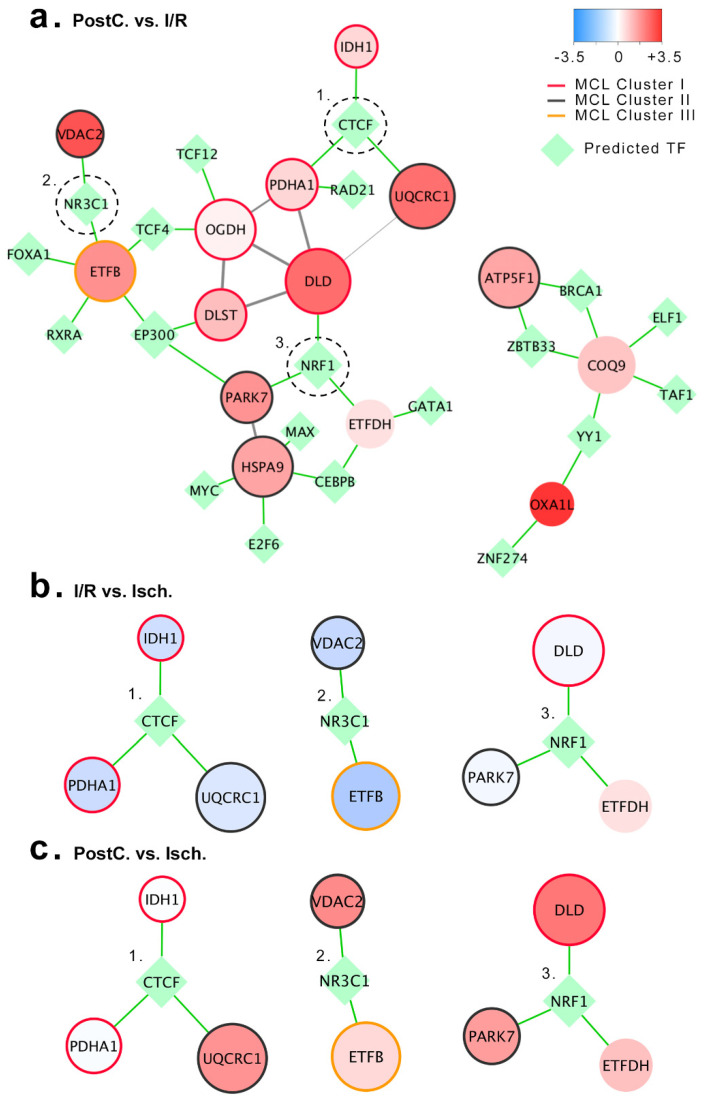
Extension of the post-conditioning mitochondrial protein-protein interaction (PPI) network with the ENCODE human regulatory data. Only proteins exhibiting an absolute logFC > 0.5 at post-conditioning versus I/R, were included. (**a**) Mitochondrial post-conditioning associated PPI network including regulatory elements retrieved from the ENCODE project human regulatory data. Nodes with no prediction of regulatory elements were hided, to simplify visualization. Nodes fill color represents logFC between post-conditioning and I/R and size represents node degree within the complete network Predicted regulatory elements are indicated as green diamonds. (**b**) Regulatory sub-network of CTCF, NR3C1, and NRF1 at ischemia/reperfusion compared to ischemia. (**c**) Regulatory sub-network of CTCF, NR3C1, and NRF1 at post-conditioning compared to ischemia. Abbreviations—Isch: Ischemia group; I/R: Ischemia/revascularization group; PostC: Post-conditioning group; TF: Transcription factor; ATP5F1: ATP synthase beta subunit; BRCA1: Breast cancer type 1 susceptibility protein; CEBPB: CCAAT/enhancer-binding protein beta; COQ9: Ubiquinone biosynthesis protein COQ9; CTCF: CCCTC-binding factor; DLD: Dihydrolepolyl dehydrogenase; DLST: Dihydrolipollysin-residue succinyltransferase component of 2-oxoglutarate dehydrogenase complex; E2F6: Transcription factor E2F6; ELF1: ETS-related transcription factor Elf-1; EP300: Histone acetyltransferase p300; ETFB: Electron transfer flavoprotein subunit beta; ETFDH: Electron transfer flavoprotein-ubiquinone oxidoreductase; FOXA1: Hepatocyte nuclear factor 3-alpha; GATA1: Erythroid transcription factor; HSPA9: Stress-70 protein, mitochondrial; IDH2: Isocitrate dehydrogenase [NADPH]; MAX: Protein max; MYC: Myc proto-oncogene protein; NR3C1: Glucocorticoid receptor; NRF1: nuclear respiratory factor 1; OGDH: 2 oxoglutarate dehydrogenase; OXA1L: Mitochondrial inner membrane protein; PARK7: Protein DJ-1; PDHA1: Pyruvate dehydrogenase; RAD21: Double-strand-break repair protein rad21 homolog; RXRA: Retinoic acid receptor RXR-alpha; TAF1: Transcription initiation factor TFIID subunit 1; TCF12: Transcription factor 12; TCF4: Transcription factor 4; UQCRC1: Cytochrome b-c1 complex subunit 1; VDAC2: Voltage-dependent anion-selective channel protein 2; YY1: Transcriptional repressor protein YY1; ZBTB33: Transcriptional regulator Kaiso; ZNF274: Neurotrophin receptor-interacting factor homolog.

In order to better understand the gene regulation at play following post-conditioning, we expanded the PPI network built upon the post-conditioning differentially expressed genes compared to direct revascularization with the human regulatory transcription factor network data retrieved from the ENCODE [38] database. As a result, 20 transcription factors were predicted to be at play (Figure 5a). From them all, the CCCTC-binding factor (CTCF), the glucocorticoid receptor (NR3C1), and the nuclear respiratory factor 1 (NRF1) were connected to nodes exhibiting opposite regulation at revascularization and post-conditioning versus ischemia (Figure 5b,c).

## 3. Discussion

### 3.1. Mitochondrial Response to Ischemia in the Pig Heart

In aerobic conditions, heart mitochondria rely on three main metabolic pathways to satisfy the ATP demand. These are the fatty acid β-oxidation (FAO), the TCA cycle, and the OXPHOS system. During FAO, fatty acids are sequentially oxidized and ultimately converted to reducing equivalents (NADH and FADH2), acetyl-CoA, and GTP (through substrate-level phosphorylation). Then, acetyl-CoA enters the TCA cycle where it is further oxidized, thereby producing more reducing equivalents, GTP, and CO_2_. In the course of OXPHOS, all reducing equivalents are utilized by the electron transport chain (ETC) complexes to pump protons into the inter-membrane space, generating an electrochemical gradient that will drive the main ATP production, supported by the F_1_F_o_-ATP-synthase (complex V). During OXPHOS, electrons released from the oxidation of reducing equivalents flow through the ETC, ultimately reaching the cytochrome-c oxidase (complex IV), where O_2_ acts as the final electron acceptor, and H_2_O is produced.

During ischemia, O_2_ deprivation rapidly inhibits the electronflow through the ETC, leading to the accumulation of reducing equivalents. The catabolic metabolism strictly depends on the re-oxidation of reducing equivalents to progress, as they exist in a relatively low abundance in the cell and cannot be imported [31]. The ETC inhibition directly impacts the TCA cycle progression and FAO pathway. In the absence of O_2_, ATP can no longer be produced by OXPHOS; instead, metabolism switches to anaerobic glycolysis, which is virtually the only way of producing ATP in the absence of O_2_. Whilst the lack of oxidized reducing equivalents disrupts the normal TCA cycle progression, some TCA cycle intermediates accumulate during a period of oxygen deprivation as a result of non-canonical TCA cycle activity [39,40,41]. This non-canonical flow through the TCA cycle leads to the accumulation of succinate but also guarantees a second source of ATP in the absence of O_2_. Only a few changes in the mitochondrial proteome were found following ischemia; these were the down-regulation of the TCA cycle enzymes DLD and SUCLA2 and the ETC members MT-ND2 and ATP5A1.

### 3.2. Mitochondrial Response to Revascularization in the Pig Heart

At the onset of revascularization, O_2_ availability restores the normal ETC activity. When this happens, succinate that has been accumulating during ischemia is oxidized at ETC complex II, reducing the ubiquinone pool and increasing the mitochondrial potential. These two conditions, together with a compromised ATP-synthase activity due to a low ADP availability following ischemia, promote reverse electron transport (RET) through complex I, ending up in the generation of reactive oxygen species (ROS) [42,43]. Despite ROS being continuously produced during metabolic activity, there is a balance with the endogenous antioxidant mechanisms, as they have the potential to induce several macromolecular alterations ranging from protein misfolding to DNA damage. Yet, ROS have a central role in myocardial damage following revascularization [44,45]. Within complex I, the mitochondrial encoded subunit ND2 (MT-ND2) and flavin mononucleotide-binding subunits are the most susceptible to oxidative damage, as they comprise the main superoxide generation site during RET [46]. Oxidative damage to complex I following revascularization is reflected in the swine mitochondrial proteome as the down-regulation of both MT-ND2 and the NADH dehydrogenase (ubiquinone) flavoprotein 1 (NDUFS1). RET through complex I is dependent on a high mitochondrial potential and a reduced ubiquinone pool and is thus transitory [47]. Yet, complex III has been identified as the second major source for ROS generation at the mitochondria as a result of electron leakage [48,49]. In contrast to the RET-driven ROS generation by complex I, complex III ROS generation occurs even under physiologic conditions [49] and is promoted following the re-establishment of coronary blood flow [47]. Consistently, a strong down-regulation of complex III was found following revascularization, which may be a consequence of oxidative damage to the ETC [50].

In addition to a compromised ETC, the expression of several enzymes belonging to the TCA cycle was found to be affected by ischemia and revascularization. This is the case of SUCLA2 and DLD, which were down-regulated during ischemia and not recovered after revascularization, and the isocitrate dehydrogenase components IDH3A and IDH2, which were found to be down-regulated after the re-establishment of coronary blood flow. The oxoglutarate dehydrogenase complex component OGDH is the only TCA cycle enzyme that was up-regulated at revascularization.

### 3.3. Mitochondrial Response to Post-Conditioning in the Pig Heart

The application of post-conditioning at the onset of revascularization had a great impact on the mitochondrial proteome of the myocardium at risk. When post-conditioning was performed, no down-regulation of complex III was detected, but robust up-regulation of complexes I, III, and V was found. These differences may result from reduced oxidative damage or de novo protein synthesis. When compared to the sham group, all the complex I members MT-ND2, NDUFSV1, and the complex III subunit UQCRC1, were seen to be strongly up-regulated, suggesting de novo synthesis (Supplemental Figure S2). Consistently, OXA1L, a protein involved in the insertion and correct assembling of inner mitochondrial membrane integral proteins, was greatly up-regulated following post-conditioning (both compared to direct revascularization and sham groups). Conversely, the complex III subunit UQCRFS1, and the complex V subunits ATP5A1 and ATP5F1, remained unaltered when compared to sham but were down-regulated after direct revascularization. These results are in line with previous studies performed in ex vivo Langerdoff-perfused rodent hearts [51,52], thus confirming the pivotal implication of the ETC complexes in the cardioprotection conferred by post-conditioning in a pre-clinical animal model of myocardial infarction. Moreover, ETFB and ETFDH, the links between the ETC and FAO, were found to be up-regulated in the post-conditioned hearts, indicating a higher implication of FAO in the post-ischemic cardiac metabolism in the post-conditioned hearts.

It is worth noting that whilst revascularization led to the down-regulation of the isocitrate dehydrogenase components IDH3A and IDH2, together with the non-recovery of SUCLA2 and DLD, post-conditioning induced a wide up-regulation of the enzymes involved in the progression of the TCA cycle, suggesting that post-conditioning promotes a canonical TCA cycle progression.

Changes in the mitochondrial proteome reflect the balance existing between metabolic adaptation and damage. Amongst the non-metabolic differentially expressed proteins detected in the post-conditioned hearts, both VDAC2 and DJ-1 have been previously related to cardioprotection [53,54,55]. Furthermore, HSPA9, a protein necessary for the mitochondrial import of DJ-1 [56] was also up-regulated. Additionally, VDAC2, DJ-1, and HSPA9 exhibited strong up-regulation when compared to the sham group, supporting the induction of a cardioprotective program following post-conditioning (Supplemental Figure S3). Yet the exact role that these proteins play in the response to ischemia and revascularization remains to be investigated.

Network biology provides an intuitive and powerful approach to characterize and understand cellular mechanisms and pathology. As shown here, network topology tightly correlates with functional modules, and collectively highlights the great implications of the energy metabolism in the cardioprotection conferred by post-conditioning. Furthermore, networks can be expanded by adding new pairs of scientifically proven interactions arising from pre-built interaction networks, allowing the identification of protein complexes and regulatory elements. In such a way, the ENCODE project includes the information of validated chromatin immunoprecipitation sequencing data sets for over 119 distinct transcription factors [38]. The post-conditioning associated PPI mitochondrial network extension through the human regulatory data retrieved from the ENCODE project revealed 20 transcription factors putatively at play in the regulation of the post-conditioning response. Amongst them, CTCF, NR3C1, and NRF1 exhibited the most consistent topology with the post-conditioning proteomic response and may be new targets for cardioprotective strategies. Further research is needed in order to elucidate the transcriptional regulation at play and upstream signaling.

## 4. Materials and Methods

### 4.1. Experimental Model

Twenty-one regular farm pigs were randomized into four experimental groups: (I) closed-chest 90 min left anterior descending (LAD) coronary artery balloon occlusion with no revascularization (ischemia group; N = 7); (II) closed-chest 90 min LAD occlusion followed by 2.5h of revascularization (ischemia-revascularization group; N = 5); (III) closed-chest 90 min LAD occlusion followed by post-conditioning and 2.5 h of revascularization (post-conditioning group; N = 5); and (IV) sham-operated animals, which underwent the same surgical procedure without balloon inflation (sham group; N = 4). To avoid thrombotic complications due to catheter manipulation, a loading dose of clopidogrel was administered to all animals 12h before the experimental procedure. Closed-chest LAD occlusion was performed as previously described under angiographic monitoring [33]. Briefly, anesthesia was administered by an intramuscular injection of zoletil^®^ (7mg/Kg), domtor^®^ (7mg/Kg), and atropine (0.03mg/Kg). Then, animals underwent endotracheal intubation, and anesthesia was maintained by isofluorane inhalation (2%). Continuous infusion of amiodarone (300mg, 75mg/h) was initiated at the beginning of the procedure in all pigs as prophylaxis for malignant ventricular arrhythmias. These amiodarone doses do not alter hemodynamic parameters [57]. Angiography was employed both to guide angioplasty balloon placement (below the first diagonal branch) and to corroborate the successful occlusion of the LAD coronary artery (no flow downstream balloon position upon contrast injection). Animals were then randomized to one of the four groups described (Supplemental Figure S1). The post-conditioning protocol consisted of six cycles of 20 s of balloon disinflation (revascularization) and 20 s of re-occlusion (ischemia) at the onset of revascularization [58,59]. Animals were not allowed to recover from the anesthesia.

Heart rate and electrocardiogram were monitored throughout the experimental procedure. Left ventricular ejection fraction (LVEF) was assessed at baseline, 90 min post-LAD occlusion (before revascularization), and at the end of revascularization time (sacrifice). All echocardiographic measurements were taken blindly by the same technician.

### 4.2. Sample Collection and Protein Extraction

Evan’s blue dye was injected in anesthetized animals to outline the area at risk, immediately after hearts were arrested, rapidly excised, and sliced. Slices were alternatively collected for infarct size analysis with triphenyl tetrazolium chloride (TTC) and sample collection. Heart samples from the inner border zone of the area at risk were collected, snap-frozen, smashed to powder, and homogenized in urea/thiourea buffer for proteomic analysis. The protein concentration was then quantified with 2D-Quant Kit (GE Healthcare, Chicago, IL, USA).

### 4.3. Proteomic Analysis

Protein extracts were separated by two-dimensional gel electrophoresis as previously described [53]. Gels were then labeled with Flamingo Fluorescent Gel Stain (Bio-Rad, Hercules, CA, USA), scanned in a Typhoon 9400 (GE Healthcare, Chicago, IL, USA), and analyzed for spot variations using PD-Quest 8.0 (Bio-Rad, Hercules, CA, USA). Three animals from each group were analyzed. Spots of interest were then excised and identified by matrix-assisted laser desorption/ionization-time-of-flight using an AutoFlex III Smartbeam MALDI-ToF/ToF (Bruker Daltonics, Billerica, MA, USA).

### 4.4. In Silico Analysis

Spot intensities in each gel were normalized by the intensity of the albumin spot. Intensity medians were then used to calculate the log-fold change (logFC) between groups, and only those proteins exhibiting an absolute logFC higher than 0.5 were considered to be differentially expressed. Differentially expressed proteins were employed to build a PPI network according to the STRING [34] database. The resulting network was then imported to Cytoscape 3.0 [60] for further analysis and visualization. The MCL strategy with an inflation parameter of 1.4 was applied in order to predict functional clusters [35,36]. Seed nodes were analyzed for functional enrichment in Gene Ontology biological process terms and pathways from Wikipathways [37] database. In order to predict putative transcription factors at play, the differentially expressed PPI network was expanded according to the human regulatory network derived from the ENCODE [38] database using the Cytoscape app CyTargetLinker [61].

### 4.5. Statistical Analysis

A bootstrapping approach was employed to calculate the effect size of each condition amongst the identified proteins. Briefly, a random sampling with replacement step was performed upon normalized spot intensities before logFC calculations [62]. An effect size threshold for significance was set at 0.5 absolute logFC. All statistical analyses were performed with RStudio (RStudio, Boston, MA, USA). Unpaired multi-two group Gardner–Altman estimation plots were generated with the R package ‘dabester’.

## 5. Conclusions

Our systems biology analysis results collectively highlight a dual role for post-conditioning both for promoting metabolic reprogramming and a protective response potentially mediated by VDAC2 and DJ-1 in the mitochondria. Therapies targeting cardiac metabolism may thus allow the pharmacological emulation/recapitulation of post-conditioning general changes, potentially overcoming the limitations found in the clinical studies. Additionally, hallmarks of the metabolic re-adaptation were found in the post-conditioning cytoplasmic proteome as illustrated previously by the regulation of the mammalian target of rapamycin (mTOR) following post-conditioning [33]. Indeed, cardiac metabolism has been suggested as a driver for cardioprotection, and several metabolic approaches have been proposed as beneficial within the AMI framework [63]. Our results describe the complex mitochondrial proteomic signature triggered by post-conditioning. Further research is needed in order to explore the upstream regulation and the link existing between VDAC2, DJ-1, and the post-ischemic metabolic adaptation to revascularization.

## Figures and Tables

**Table 1:** Top 10 most significant Gene Ontology biological terms detected.

GO_Term	Description	Strength	FDR
GO:0006091	Generation of precursor metabolites and energy	1.59	2.88 × 10^−25^
GO:0045333	Cellular respiration	1.9	1.24 × 10^−23^
GO:0055114	Oxidation-reduction process	1.2	6.17 × 10^−17^
GO:0009060	Aerobic respiration	2	2.41 × 10^−14^
GO:0006099	Tricarboxylic acid cycle	2.27	6.75 × 10^−13^
GO:0044281	Small molecule metabolic process	0.92	4.85 × 10^−11^
GO:0022904	Respiratory electron transport chain	1.77	2.40 × 10^−9^
GO:0006119	Oxidative phosphorylation	1.72	4.49 × 10^−9^
GO:0046034	ATP metabolic process	1.54	5.51 × 10^−9^
GO:0019752	Carboxylic acid metabolic process	1.08	1.14 × 10^−8^

## Data Availability

The data presented in this study are available in supplementary material.

## Supplementary Materials

The following supporting information can be downloaded at: https://www.mdpi.com/article/10.3390/ijms23042087/s1.
