# Supplementary material for: Network-Assisted Systems Biology Analysis of the Mitochondrial Proteome in a Pre-Clinical Model of Ischemia, Revascularization and Post-Conditioning
Source: Int J Mol Sci. 2022 Feb 14;23(4):2087. doi: 10.3390/ijms23042087 (PMC8879554; doi:10.3390/ijms23042087)

Table S1. Changes in mitochondrial proteins detected in the swine at-risk myocardial proteome.

Protein name	Symbol	Isch vs. Sham	I/R vs. Isch	PostC vs. Isch	PostC vs. I/R
Voltage-dependent anion-selective channel protein 1	VDAC1	↓ -1.84	N.D.	↑ 3.45	N.D.
D-beta-hydroxybutyrate dehydrogenase	BDH1	→ 0.41	N.D.	↑ 2.48	N.D.
Mitochondrial inner membrane protein	OXA1L	↓ -0.57	↓ -1.87	↑ 1.66	↑ 3.53
Voltage-dependent anion-selective channel protein 2	VDAC2	→ 0.04	↓ -0.94	↑ 2.08	↑ 3.02
Dihydrolepolyl dehydrogenase	DLD	↓ -1.95	→ -0.18	↑ 2.40	↑ 2.58
Cytochrome b-c1 complex subunit 1	UQCRC1	→ -0.12	↓ -0.59	↑ 1.95	↑ 2.54
NADH-ubiquinone oxidoreductase	MT-ND2	↓ -0.60	→ -0.30	↑ 2.16	↑ 2.46
Electron transfer flavoprotein subunit beta	ETFB	↑ 0.68	↓ -1.28	↑ 0.70	↑ 1.98
Protein DJ-1	PARK7	↑ 0.62	→ -0.19	↑ 1.74	↑ 1.93
NADH dehydrogenase [ubiquinone] flavoprotein 1	NDUFV1	→ -0.15	↓ -0.58	↑ 1.30	↑ 1.87
Stress-70 protein, mitochondrial	HSPA9	↓ -0.67	→ -0.24	↑ 1.37	↑ 1.62
ATP synthase beta subunit	ATP5F1	→ -0.06	↓ -1.04	↑ 0.58	↑ 1.61
Succinyl-CoA:3-ketoacid-coenzyme	OXCT1	→ 0.01	→ -0.39	↑ 1.08	↑ 1.47
Cytochrome b-c1 complex subunit Rieske	UQCRFS1	→ 0.40	↓ -1.36	→ 0.10	↑ 1.46
Dihydrolipollysin-residue succinyltransferase component of 2-oxoglutarate dehydrogenase complex	DLST	→ -0.29	→ 0.03	↑ 1.21	↑ 1.17
Hydroxyacyl-coenzyme A dehydrogenase	HADH	→ 0.33	↓ -1.06	→ 0.07	↑ 1.13
Ubiquinone biosynthesis protein COQ9	COQ9	→ -0.24	→ -0.29	↑ 0.75	↑ 1.04
Isocitrate dehydrogenase [NAD] subunit alpha	IDH3A	→ -0.42	↓ -0.98	→ 0.01	↑ 0.99
Succinyl-CoA ligase [ADP-forming] subunit beta	SUCLA2	↓ -0.57	→ -0.27	↑ 0.51	↑ 0.78
Pyruvate dehydrogenase	PDHA1	→ 0.18	↓ -0.84	→ -0.08	↑ 0.75
Isocitrate dehydrogenase [NADPH]	IDH2	↑ 0.66	↓ -0.77	→ -0.05	↑ 0.72
Heart aconitase	ACO2	→ 0.12	→ 0.07	↑ 0.72	↑ 0.65
ATP synthase subunit alpha	ATP5A1	↓ -0.70	→ -0.08	↑ 0.54	↑ 0.63
Electron transfer flavoprotein-ubiquinone oxidoreductase	ETFDH	↓ -0.50	↑ 0.55	↑ 1.13	↑ 0.57
Superoxide dismutase	SOD1	→ 0.01	→ 0.15	↑ 0.52	→ 0.37
2 oxoglutarate dehydrogenase	OGDH	→ -0.31	↑ 0.52	↑ 0.77	→ 0.24

Table S2. Wikipathways enrichment analysis result of the differentially regulated proteins detected across ischemia, revascularization, and post-conditioning.

WP_id	Term description	Strength	FDR
WP2453	TCA cycle and deficiency of pyruvate dehydrogenase complex (PDHc)	2.47	2.90E-10
WP78	TCA cycle (aka Krebs or citric acid cycle)	2.42	2.90E-10
WP3925	Amino acid metabolism	1.78	1.64E-08
WP111	Electron transport chain: OXPHOS system in mitochondria	1.66	1.83E-06
WP4932	7q11.23 copy number variation syndrome	1.58	8.26E-05
WP4290	Metabolic reprogramming in colon cancer	1.87	9.45E-05
WP623	Oxidative phosphorylation	1.72	0.00031
WP311	Ketone bodies synthesis and degradation	2.5	0.0071
WP4236	Krebs cycle disorders	2.35	0.0109
WP4742	Ketogenesis and ketolysis	2.29	0.0122
WP4297	Thiamine metabolic pathways	2.24	0.0136
WP5037	Riboflavin and CoQ disorders	2.05	0.027
WP4921	Mitochondrial complex III assembly	1.99	0.0318

Figure S1. Cardioprotection conferred by post-conditioning. **A.** Left ventricular ejection fraction (LVEF) at baseline, at 90 min post AMI, and at sacrifice. (90 min post AMI and sacrifice are the same for Isch. group) **B.** Infarct size measurement expressed as a percentage of the area at risk (AAR). **C.** Scheme illustrating the experimental procedure timeline for each group. Red arrows indicate sacrifice. Data is presented as mean ± standard error. (* $p < 0.05$).

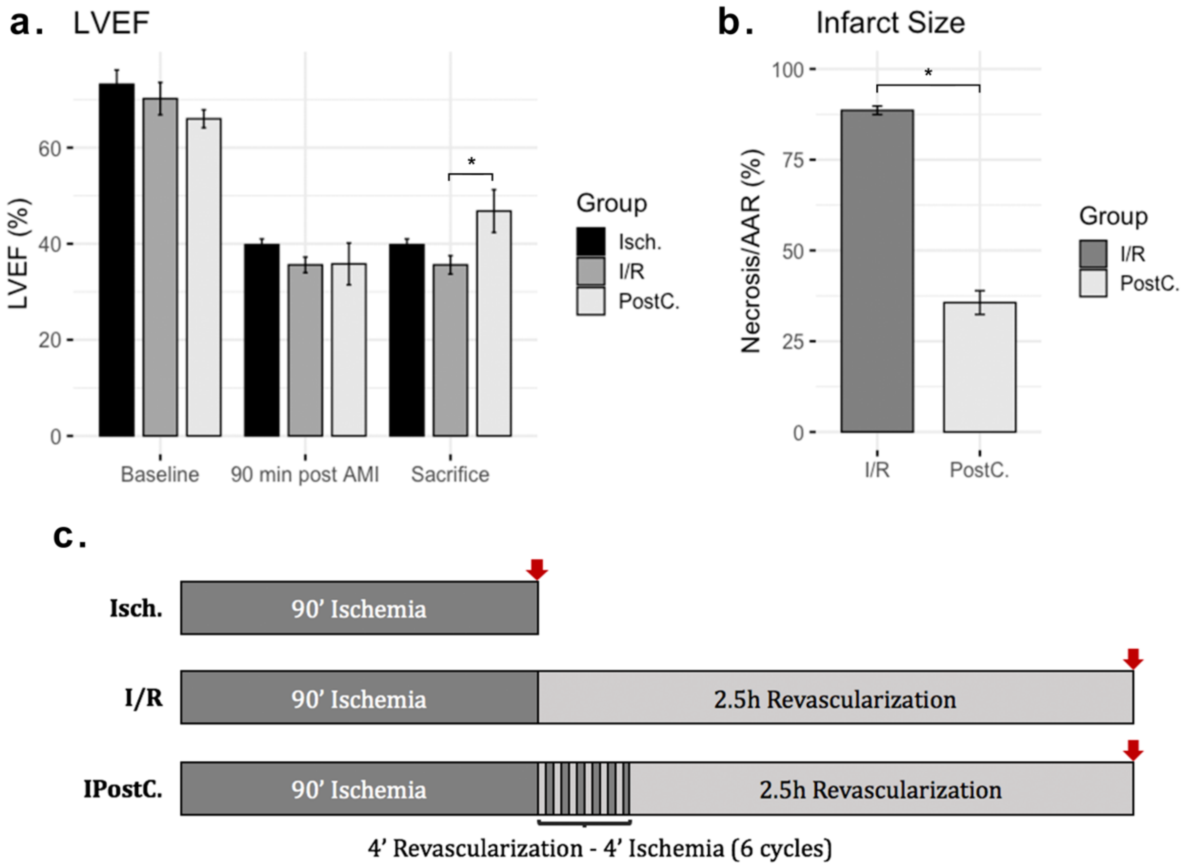

Figure S2. Multi two-group Gardner-Altman estimation plots of the ETC identified members across all conditions. The bootstrap distribution of the unpaired mean difference between each condition and the sham is depicted (gray shadow). **A.** ETC complex I. **B.** ETC complex III. **C.** ETC complex V.

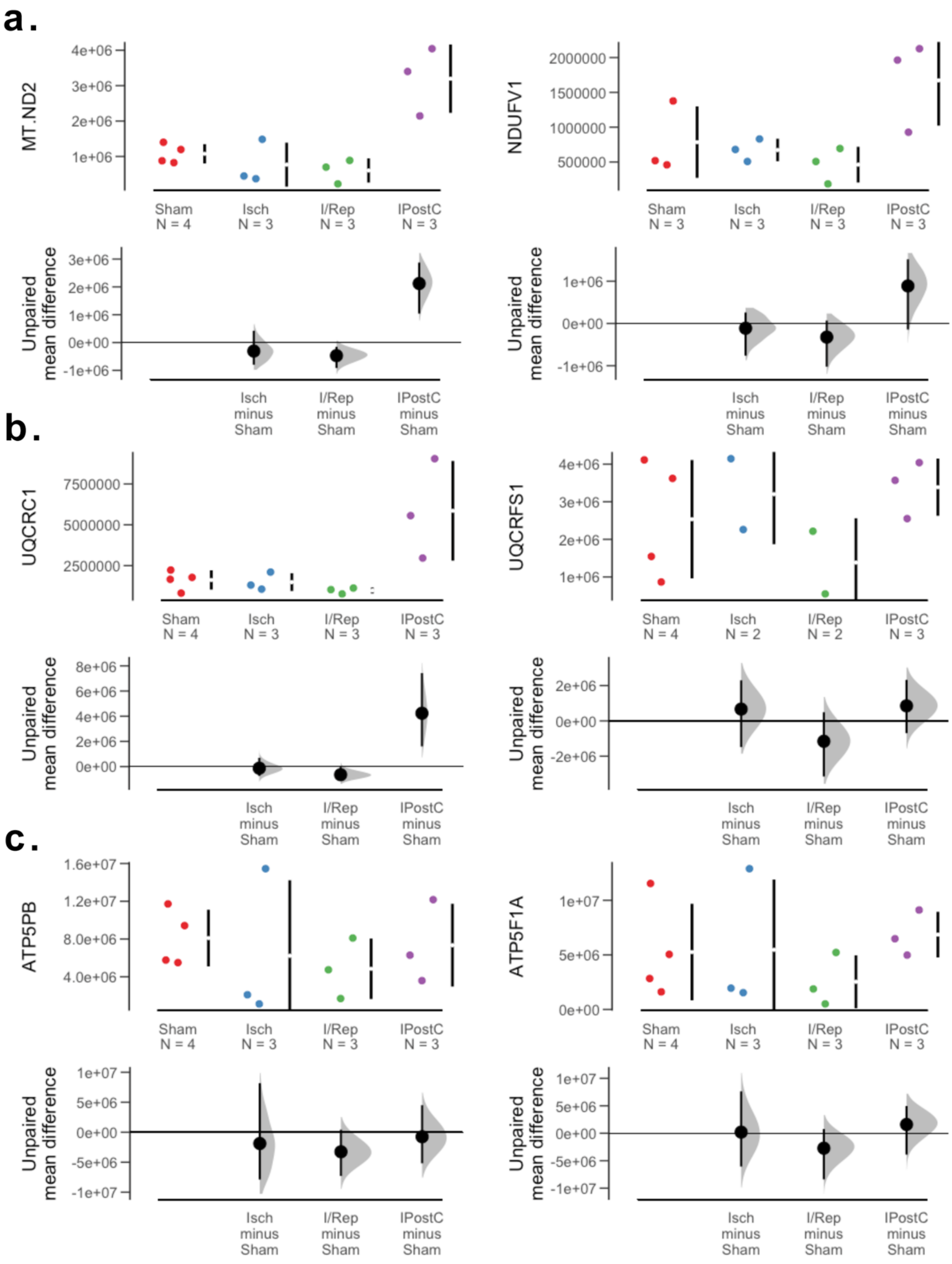

Figure S3. Multi two-group Gardner-Altman estimation plots of VDAC2, DJ-1, and HSPA9.

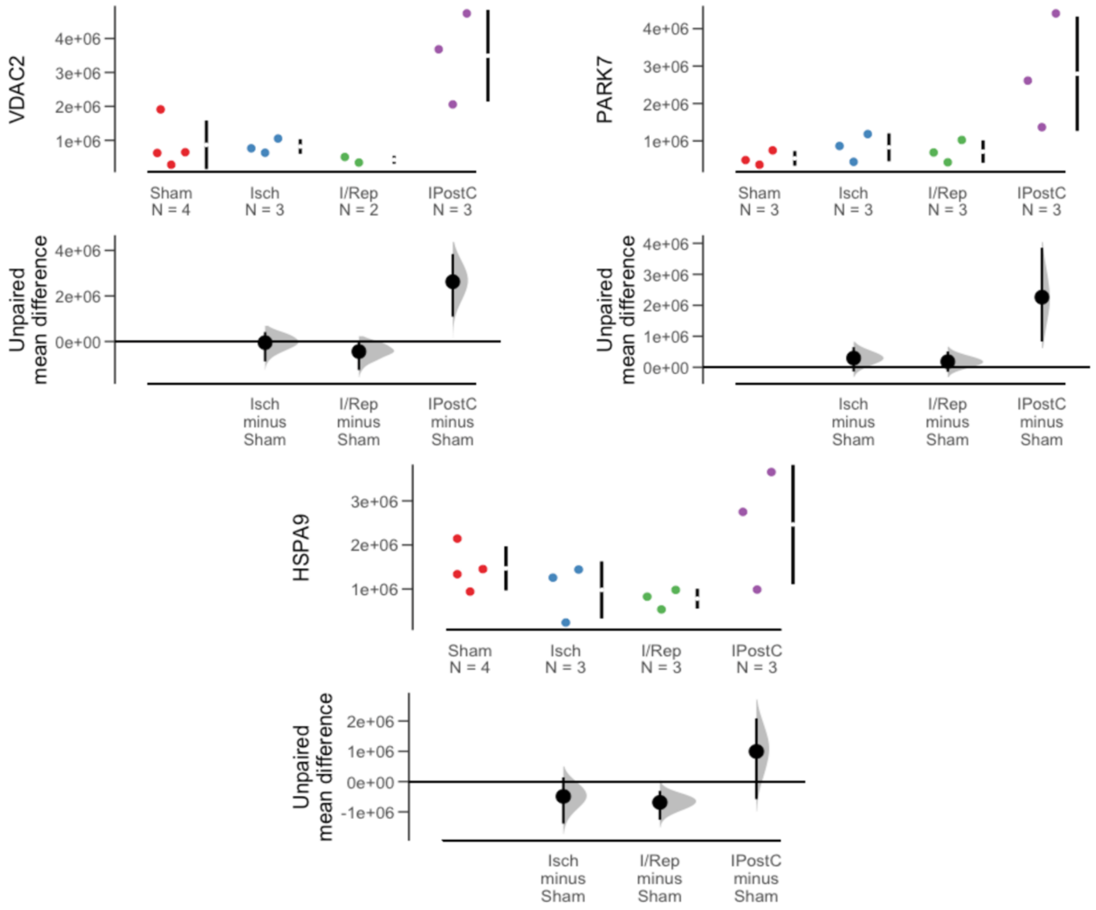

Supplement: Supplementary file 1 [file ijms-23-02087-s001.zip › ijms-1575824-supplementary.pdf]
